# Nerve growth factor promote VCAM-1-dependent monocyte adhesion and M2 polarization in osteosarcoma microenvironment: Implications for larotrectinib therapy

**DOI:** 10.7150/ijbs.95463

**Published:** 2024-08-01

**Authors:** Syuan-Ling Lin, Shang-Yu Yang, Chun-Hao Tsai, Yi-Chin Fong, Wei-Li Chen, Ju-Fang Liu, Chih-Yang Lin, Chih-Hsin Tang

**Affiliations:** 1 Translational Medicine Research Center, China Medical University Hospital, Taichung, Taiwan; 2 Department of Medical Laboratory Science and Biotechnology, Asia University, Taichung, Taiwan; 3 Department of Sports Medicine, College of Health Care, China Medical University, Taichung, Taiwan; 4 Department of Orthopedic Surgery, China Medical University Hospital, Taichung, Taiwan; 5 Department of Orthopedic Surgery, China Medical University Beigang Hospital, Yunlin, Taiwan; 6 Translational Medicine Center, Shin Kong Wu Ho-Su Memorial Hospital, Taipei, Taiwan; 7 School of Oral Hygiene, College of Oral Medicine, Taipei Medical University, Taipei City, Taiwan.; 8 Department of Pharmacology, School of Medicine, China Medical University, Taichung, Taiwan; 9 Chinese Medicine Research Center, China Medical University, Taichung, Taiwan; 10 Department of Medical Research, China Medical University Hsinchu Hospital, Hsinchu, Taiwan

**Keywords:** osteosarcoma, NGF, larotrectinib, M2 macrophage, miR-513c-5p

## Abstract

Osteosarcoma is the most prevalent form of primary malignant bone tumor, primarily affecting children and adolescents. The nerve growth factors (NGF) referred to as neurotrophins have been associated with cancer-induced bone pain; however, the role of NGF in osteosarcoma has yet to be elucidated. In osteosarcoma samples from the Genomic Data Commons data portal, we detected higher levels of NGF and M2 macrophage markers, but not M1 macrophage markers. In cellular experiments, NGF-stimulated osteosarcoma conditional medium was shown to facilitate macrophage polarization from the M0 to the M2 phenotype. NGF also enhanced VCAM-1-dependent monocyte adhesion within the osteosarcoma microenvironment by down-regulating miR-513c-5p levels through the FAK and c-Src cascades. In *in vivo* xenograft models, the overexpression of NGF was shown to enhance tumor growth, while the oral administration of the TrK inhibitor larotrectinib markedly antagonized NGF-promoted M2 macrophage expression and tumor progression. These results suggest that larotrectinib could potentially be used as a therapeutic agent aimed at mitigating NGF-mediated osteosarcoma progression.

## Introduction

Osteosarcoma is the most common form of primary malignant bone cancer, affecting mainly children and teenagers. Men are more susceptible to the disease than women, as indicated by a male-to-female ratio of 1.5-2:1 1. Common sites for osteosarcoma include the proximal tibia (19%), proximal humerus (10%), and distal femur (42%) 2, 3. Recent advances in chemotherapy for non-metastatic osteosarcoma have boosted the five-year survival rate from 15% to 60% 4. Note however that roughly 25% of osteosarcoma patients have metastases at the time of diagnosis 5, and the prognosis in these cases remains poor. Thus, the overall five-year survival rate for patients with metastatic osteosarcoma is below 30% 6. This has prompted extensive research into the development of novel pre-treatment prognostic indicators and novel targeted medications as adjuvants to chemotherapy.

Researchers have established that malignant growth depends on interactions between tumor cells and cells within the tumor microenvironment 7, 8, which is a complex system involving a variety of cell types, including endothelial, fibroblast, and inflammatory cells (e.g., dendritic and macrophage cells) 7. Most of these cells are macrophages, specific subpopulations of which are drawn in by cytokines released by the tumor 9, 10. These tumor-associated macrophages (TAMs) can have a profound effect on tumor growth; however, the effect varies with phenotype. M1 macrophages are typically anti-tumoral, whereas M2 macrophages are typically pro-tumoral 11. TAMs have been linked to the progression and spread of cancer within the tumor microenvironment 12, 13.

Vascular cell adhesion molecule 1 (VCAM-1) plays a role in controlling the growth, migration, and death of cancer cells 14, 15. In osteosarcoma, VCAM-1 expression is correlated with tumor formation and disease stage 16. Various growth agents have been shown to promote VCAM-1 expression over the course of osteosarcoma progression and metastasis 17, 18. We hypothesize that diminishing VCAM-1 synthesis is a logical therapeutic strategy aimed at limiting osteosarcoma growth.

Tumor cells have the ability to stimulate the formation of sympathetic and sensory nerves, which can lead to cancer-induced bone pain (CIBP) 19, the treatment of which becomes increasingly difficult as the tumor grows 20. The mechanisms underlying CIBP include a variety of neuropathological events, including inflammation, ischemia, compression, or damage 21. Researchers have recently reported a strong correlation between CIBP and elevated nerve growth factor (NGF) expression levels 21. NGF is an important neurotrophic factor, which promotes the development of sympathetic and sensory nerves. It is abundantly expressed in the tumor microenvironment, where it interacts with various cell types to facilitate cancer progression 22. NGF is catalyzed by the high-affinity receptor tropomyosin receptor kinase A (TrkA), which is a kinase shown to regulate tumor growth, neuronal differentiation, neural proliferation, and nociceptor responsiveness as well as prevent programmed cell death 22, 23. The NGF/TrkA axis has been implicated in the metastasis of various cancers, including breast cancer 24, chondrosarcoma 25, colon cancer 26, pancreatic cancer 27, and prostate cancer 28. Studies have shown that blocking NGF or TrkA expression can significantly slow the growth of tumors, reduce pain-related activities, and ameliorate bone loss in sarcoma patients 25, 29.

Researchers in the field of medicinal chemistry have made considerable strides in elucidating TrK inhibitors. Larotrectinib (LOXO-101) is a highly specific small molecule inhibitor of the entire TrK family, including TrkA, TrkB, and TrkC. The FDA has approved its usage in the treatment of malignancies that are TRK fusion-positive, including lung 30 and breast 31 cancer. In the current study, we determined that NGF facilitates VCAM-1-dependent monocyte adhesion and M2 polarization in the osteosarcoma microenvironment. We also determined that larotrectinib inhibits NGF-mediated effects *in vitro* and *in vivo*, which suggests the potential of using larotrectinib to develop novel therapeutic agents for osteosarcoma.

## Materials and Methods

NGF recombinant protein was obtained from PeproTech (Rocky Hill, NJ, USA). p-FAK (Tyr397) (Catalog No: #3283) and p-c-Src (Tyr416) (Catalog No: #2101) antibodies were obtained from Cell Signaling (Danvers, MA, USA). FAK (Tyr397) (GTX129840) antibodies were purchased from GeneTex (Hsinchu, Taiwan). c-Src (Catalog No: 25978-1-AP), CD163 (Catalog No: 16646-1-AP), and CD206 (Catalog No: 18704-1-AP) antibodies were purchased from Proteintech (Rosemont, IL, USA). VCAM-1 (EPR5047) (Catalog No: ab134047) and NGF (EP1320Y) antibodies (Catalog No: ab52918) were obtained from Abcam (Cambridge, MA, USA). β-actin antibodies (Catalog No: a5441) were obtained from Sigma-Aldrich (St. Louis, MO, USA). Cell culture supplements were obtained from Invitrogen (Carlsbad, CA, USA). siRNAs against VCAM-1, FAK, c-Src, and controls were purchased from Dharmacon (Lafayette, CO, USA). All other reagents were obtained from Sigma-Aldrich (St. Louis, MO, USA).

### Analysis of mRNA expression profiles from the Genomic Data Commons (GDC)

Transcriptome profiles of osteosarcoma were accessed using the TCGA database via the GDC database. RNA-Seq analysis was performed on osteosarcoma samples to derive gene expression profiles of macrophage markers and the neurotrophin family 32.

### Cell cultures

Human osteosarcoma cell lines 143B and MG63 and the human monocyte cell line THP-1 were obtained from the American Type Culture Collection (Manassas, VA, USA). The cells were cultured in Dulbecco's Modified Eagle Medium (DMEM) containing 10% FBS, penicillin, and streptomycin at 37°C under a humidified atmosphere of 5% CO_2_ 33.

THP-1 cells were differentiated into M0 macrophages via stimulation with 100 ng/ml phorbol 12-myristate 13-acetate (PMA) for 24 h followed by incubation in DMEM medium for 24 h.

### Analysis monocyte adhesion to osteosarcoma

Osteosarcoma cells (1 × 10^5^) were seeded in 12-well plates and allowed to reach a confluency of roughly 90%. The osteosarcoma cells were then exposed to NGF for 24 h. Monocytes were stimulated via exposure to BCECF-AM (10 μM) for 1 h, followed by co-culturing with osteosarcoma cells for 1 h. Non-adherent monocytes were then washed away, and adherent monocytes were quantified using a fluorescence microscope 34.

### MicroRNA (miRNA) database searches

We employed the miRNA database miRWalk: Home (http://mirwalk.umm.uni-heidelberg.de/) to predict miRNAs that could potentially target the VCAM-1 gene. Incorporating the miRDB database with a filter set to a minimum threshold of 0.8 resulted in the identification of 14 miRNAs with potential binding affinity to the VCAM-1 gene.

### Real-time quantitative PCR analysis of mRNA and miRNA

Total RNA was extracted from osteosarcomas using TRIzol reagent (MDBio; Taipei, Taiwan). Subsequently, 1 μg of RNA was reverse-transcribed into cDNA using an oligo-DT primer, in accordance with the protocol outlined by the manufacturer (Invitrogen; Carlsbad, CA, USA). Quantitative PCR (qPCR) was performed using SYBR Green with sequence-specific primers (Invitrogen; Carlsbad, CA, USA). qPCR assays were performed using the StepOnePlus system (Applied Biosystems; Foster City, CA, USA) 35, 36. The sequences of all primers are listed in Table 1.

### Western blot analysis

Protein samples extracted from osteosarcoma cells using RIPA buffer underwent electrophoretic separation using SDS-PAGE gels (7.5-12%) prior to transfer onto PVDF membranes (Merck; Darmstadt, Germany). After blocking with 5% non-fat milk, the membranes were exposed to primary antibodies at 4°C overnight and then exposed to specific secondary antibodies at room temperature for 1 h. Target protein expression was detected using an ECL kit (Millipore, USA) and visualized using the ImageQuant™ LAS 4000 biomolecular imager 37-39. Bands were subsequently digitized using UN-SCAN-IT gel 6.1 software. Graphs were generated using GraphPad Prism after normalizing the data using Microsoft Excel.

### Mouse xenograft models

Male nude mice were obtained from BioLASCO Taiwan Co., Ltd. (Taipei, Taiwan). All procedures involving animal studies were granted approval by the Institutional Animal Care and Use Committee and conducted in accordance with the Guidelines of Animal Experimentation set forth by Shin Kong Wu Ho-Su Memorial Hospital (Protocol No. 113SKH007). Under 1.5-2.5% isoflurane anesthesia, 5 × 10^6^ 143B or 143B/NGF cells were subcutaneously injected into the right flank of each animal in a solution comprising 50% serum-free DMEM and 50% Matrigel (total volume of 100 μL). The 143B/NGF+Larotrectinib group was also administered larotrectinib (50 mg/kg) orally three times a week. After a four-week tumor growth period, the mice were euthanized via CO_2_ inhalation. The harvested tumors were fixed in 10% formalin in preparation for analysis 25, 40.

### Immunohistochemistry (IHC)

Immunohistochemistry assays were conducted on mouse tissues. The primary antibodies employed in immunohistochemical analysis were CD163, CD206, and VCAM-1 diluted at a ratio of 1:250. Quantification was performed in accordance with the protocol detailed in our prior publications 41, 42. Following the application of biotin-labeled secondary antibodies, the antibody binding signal was visualized using the NovoLink Polymer Detection System (Leica Biosystems Inc, IL, US) using 3,3'-diaminobenzidine with hematoxylin for counterstaining. Positive expression levels were quantified by IHC staining results, based on scores ranging from 1 (weak) to 5 (strong) 43.

### Statistical analysis

The data are presented as mean ± standard deviation (SD). Statistical analysis was performed using the two-tailed Student's t-test to determine the significance of between-group differences, with the level of significance set at 0.05.

## Results

### NGF facilitated the polarization of macrophages to the M2 phenotype in the osteosarcoma microenvironment

**Figure 1:**
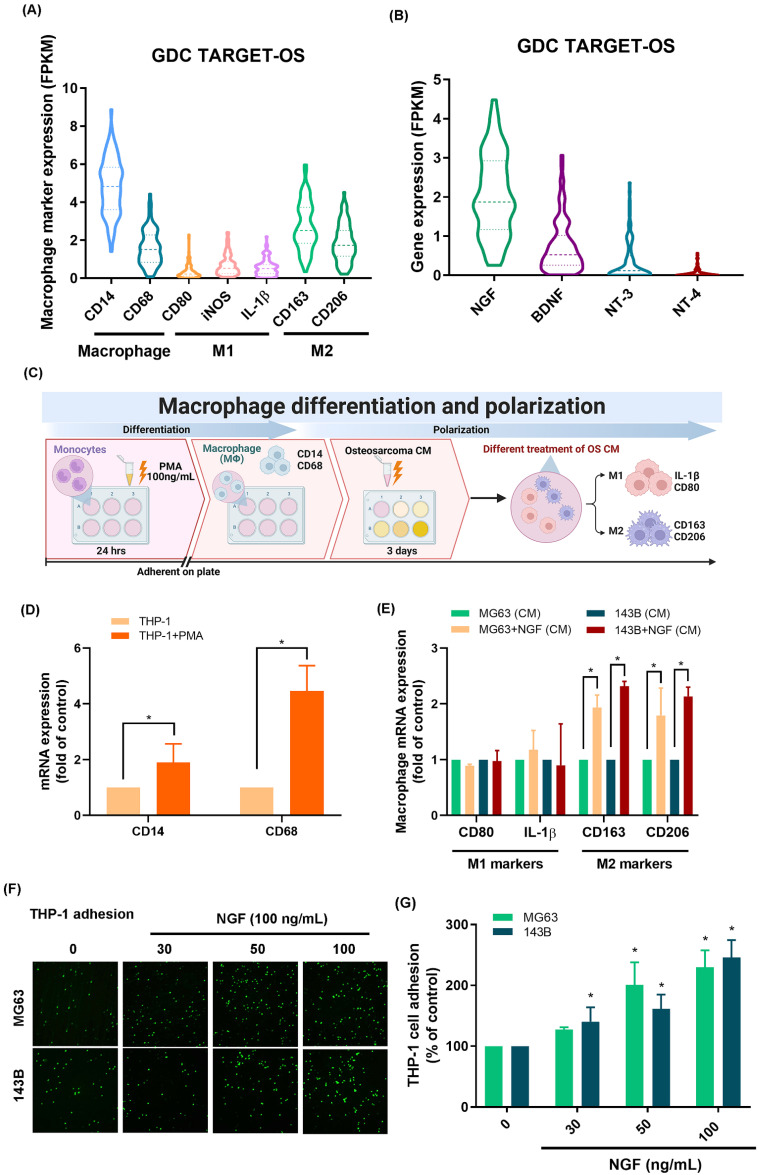
** NGF facilitates the polarization of macrophages to the M2 phenotype in the osteosarcoma microenvironment.** (A&B) mRNA expression of macrophage and neurotrophin factors in osteosarcoma tissue based on analysis of samples from the GDC data portal; (C) Schematic of Macrophage Differentiation and Polarization; (D) qPCR analysis of THP-1 cells, following incubation with PMA for 24 h; (E) qPCR analysis showing mRNA expression after treating osteosarcoma cells with NGF for 24 h and then applying conditioned medium to M0 macrophage; (F&G) Fluorescence microscope images of THP-1 cells adhered to osteosarcoma cells, following incubation with NGF for 24 h. All experiments were repeated at least three times. * *p* < 0.05 compared with the control group.

Researchers have previously demonstrated that TAMs exhibiting the M2 phenotype can accelerate tumor growth 7. Analysis of 88 osteosarcoma tissue samples from the GDC data portal revealed elevated CD14 and CD68 levels (macrophage markers) as well as the presence of CD163 and CD206 (M2 macrophage markers); however, M1 macrophage markers (CD80, iNOS and IL-1β) were not detected (Fig. 1A).

As for neurotrophin factors, it was found that the expression level of NGF mRNA exceeded those of BDNF, NT-3, and NT-4 (Fig. 1B). We then determined whether NGF promoted the polarization of M2 macrophages (Fig. 1C). Stimulating THP-1 cells with PMA resulted in the differentiation of THP-1 cells into M0 macrophages, as evidenced by the mRNA expression of M0 markers CD14 and CD68 (Fig. 1D). Exposing M0 macrophages to NGF-treated osteosarcoma conditioned medium was also shown to enhance polarization to the M2 phenotype, but not to the M1 phenotype (Fig. 1E). These findings suggest that in the osteosarcoma microenvironment, NGF enhances the polarization of naïve macrophages into the M2 phenotype.

### NGF facilitated VCAM-1-dependent monocyte adhesion to osteosarcoma

**Figure 2:**
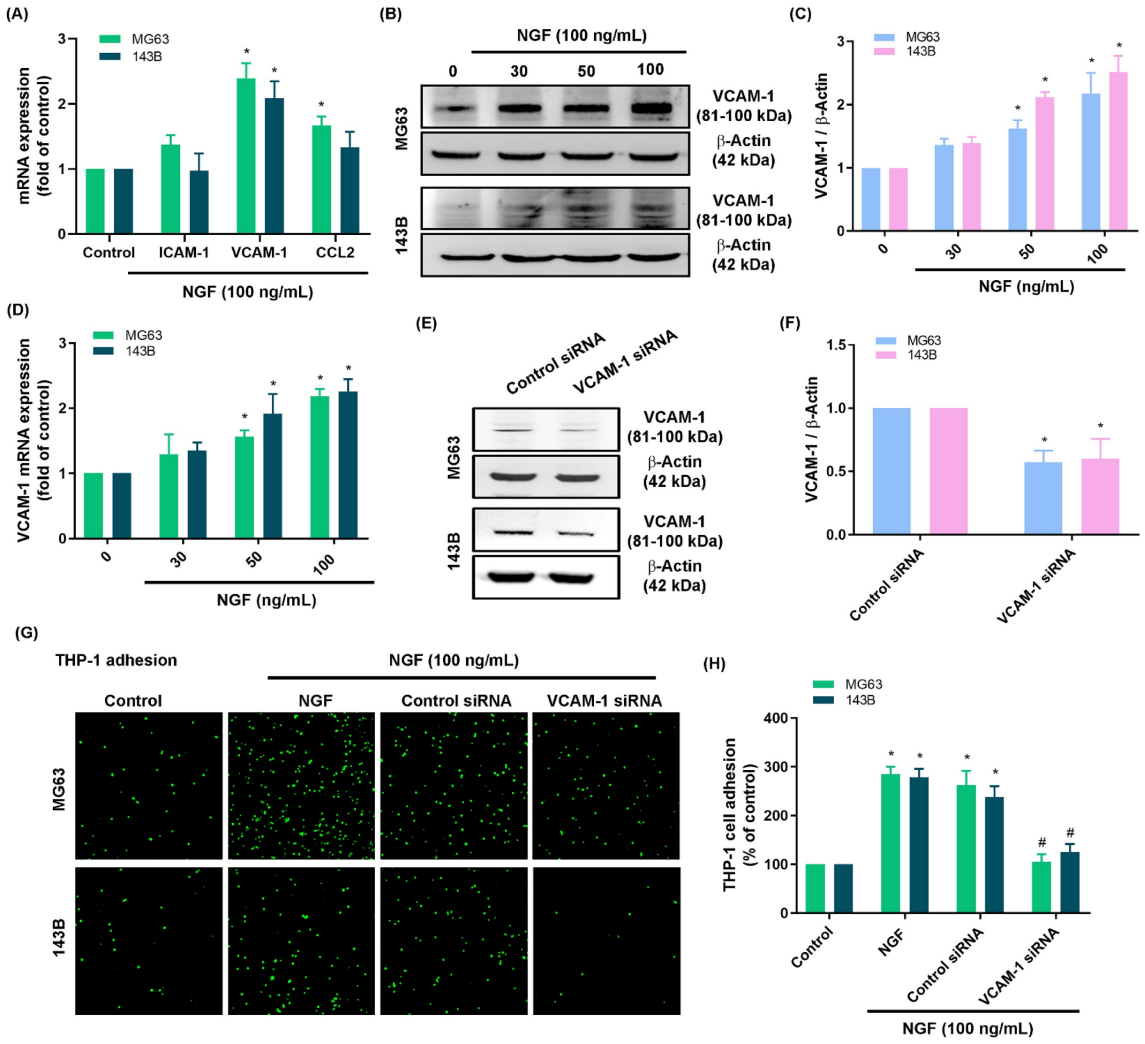
** NGF enhances VCAM-1 generation and monocyte adhesion to osteosarcoma.** (A) qPCR results indicating mRNA expression after treating osteosarcoma cells with NGF for 24 h; (B-D) Western blot and qPCR results indicating VCAM-1 expression after treating osteosarcoma cells with NGF for 24 h; (E&F) Western blot analysis indicating VCAM-1 expression following transfection of osteosarcoma cells with VCAM-1 siRNA; (G&H) THP-1 adhesion in osteosarcomas transfected with VCAM-1 siRNA and then stimulated with NGF for 24 h. All experiments were repeated at least three times. * *p* < 0.05 compared with the control group; # *p* < 0.05 compared with the NGF-treated group.

The infiltration of macrophages into the tumor microenvironment is a critical step in tumor proliferation and growth. Treating osteosarcoma cell lines (143B and MG63 cells) with NGF was shown to increase monocyte adhesion in a concentration-dependent manner (Fig. 1F&G). The screening of candidate targets in NGF-treated osteosarcomas revealed that the NGF-induced upregulation of VCAM-1 was more pronounced than that of ICAM-1 or CCL2 (Fig. 2A). NGF also enhanced VCAM-1 protein and mRNA expression levels in a concentration-dependent manner (Fig. 2B-D). Osteosarcoma cells transfected with VCAM-1 siRNA were shown to antagonize VCAM-1 expression and moderate NGF-induced monocyte adhesion (Fig. 2E-H), which indicates that NGF augments VCAM-1-dependent monocyte adhesion to osteosarcoma.

### NGF induced VCAM-1 production and monocyte adhesion by inhibiting miR-513c-5p expression via the FAK and c-Src pathways

**Figure 3:**
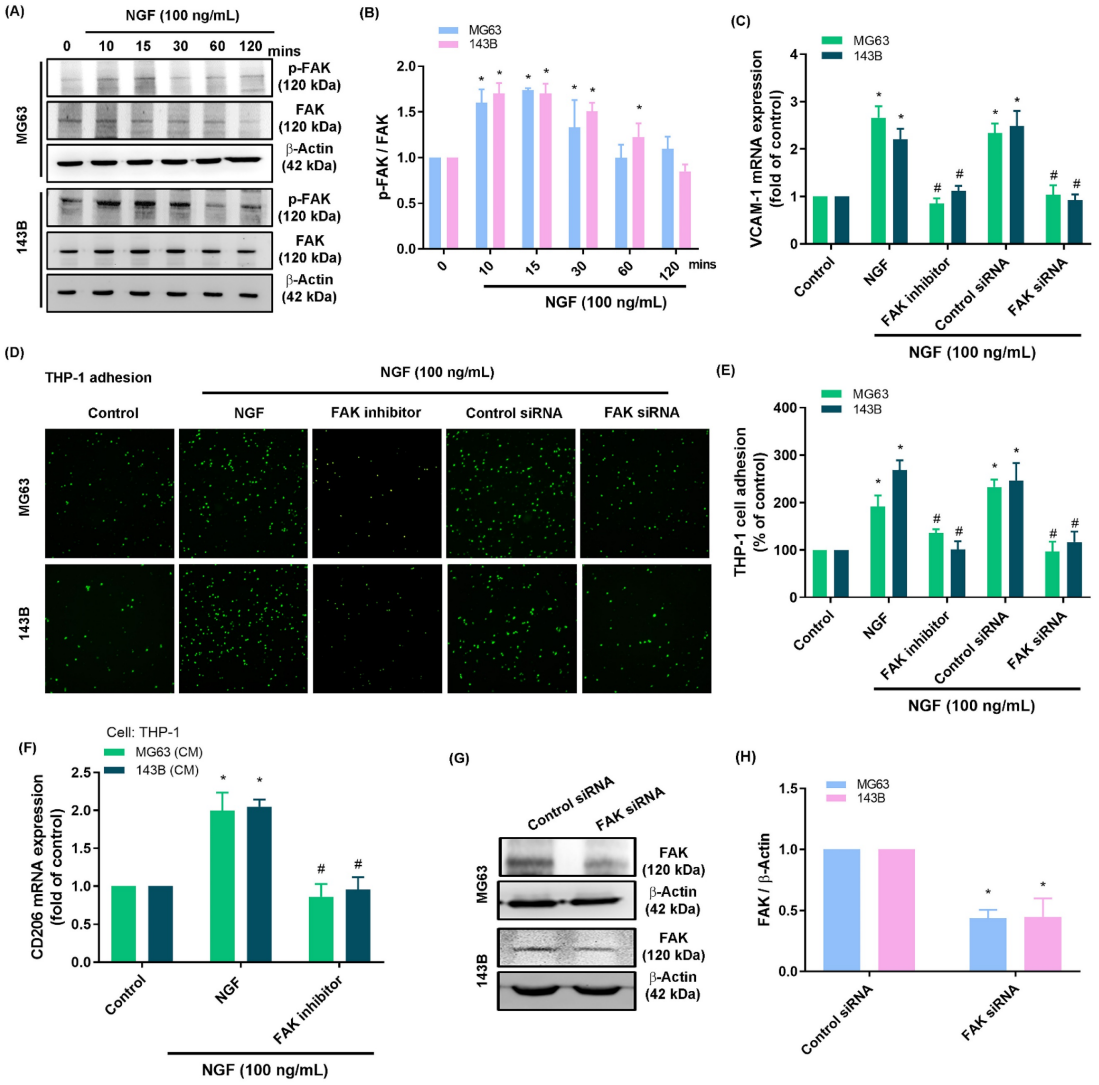
** NGF increases VCAM-1 production and monocyte adhesion to osteosarcomas via FAK signaling.** (A&B) Western blot analysis showing FAK phosphorylation in osteosarcomas stimulated with NGF; (C-E) THP-1 adhesion and VCAM-1 expression in osteosarcomas incubated with FAK inhibitor or transfected with FAK siRNA and then stimulated with NGF for 24 h; (F) The supernatant collected from osteosarcoma cells to THP-1 cells affected the expression of M2 macrophage marker CD206 mRNA expression; (G&H) Western blot analysis showing FAK expression following transfection of osteosarcoma cells with FAK siRNA. All experiments were repeated at least three times. * *p* < 0.05 compared with the control group; # *p* < 0.05 compared with the NGF-treated group.

**Figure 4:**
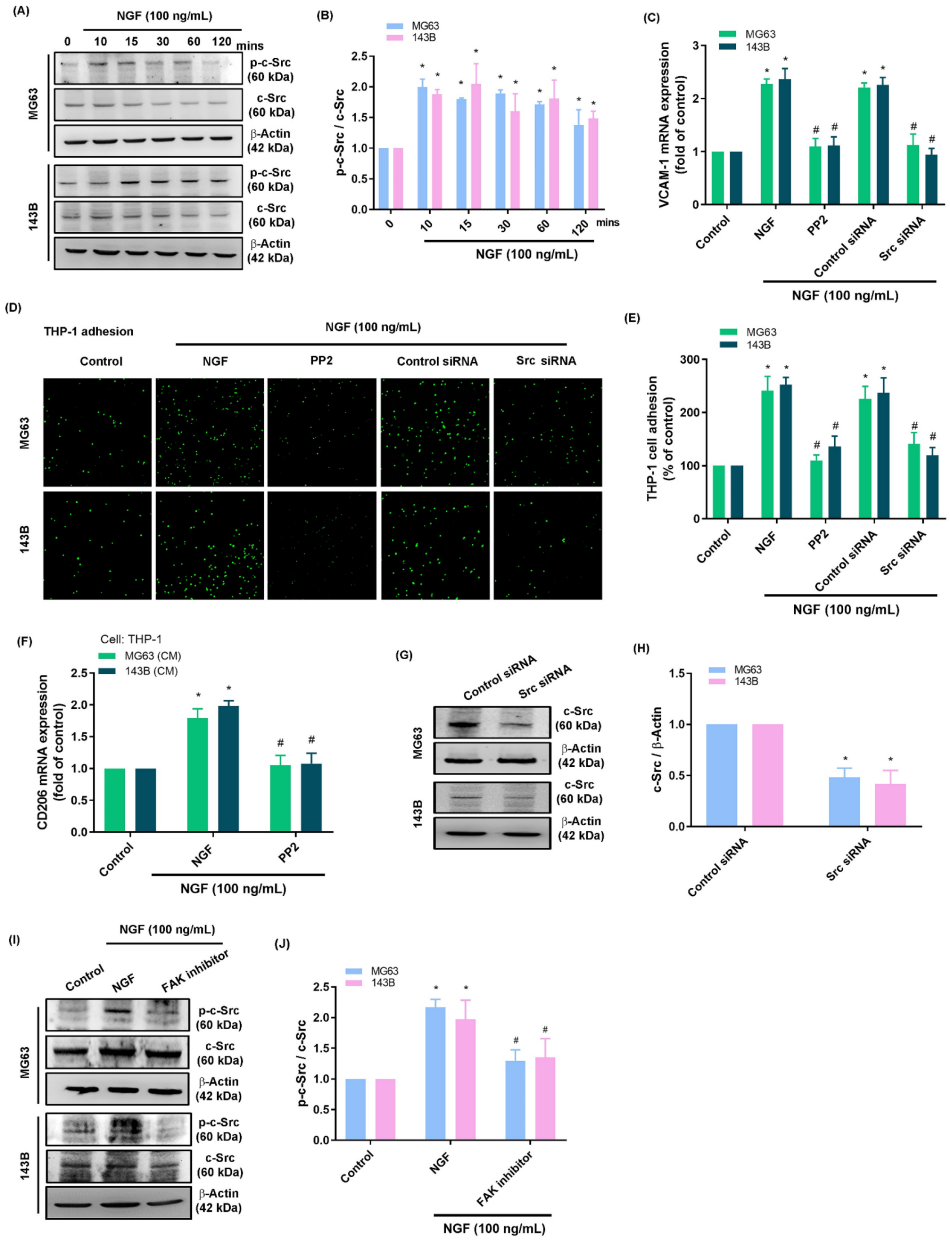
** NGF increases VCAM-1 production and monocyte adhesion to osteosarcomas via c-Src signaling.** (A&B) Western blot analysis indicating c-Src phosphorylation after stimulating osteosarcoma cells with NGF; (C-E) THP-1 adhesion and VCAM-1 expression in osteosarcoma cells incubated with PP2 or transfected with c-Src siRNA and then stimulated with NGF for 24 h; (F) The supernatant collected from osteosarcoma cells to THP-1 cells affected the expression of M2 macrophage marker CD206 mRNA expression; (G&H) Western blot analysis showing c-Src expression following transfection of osteosarcoma cells with c-Src siRNA; (I&J) Western blot analysis showing c-Src phosphorylation after incubating osteosarcoma cells with FAK inhibitor followed by stimulation with NGF. All experiments were repeated at least three times. * *p* < 0.05 compared with the control group; # *p* < 0.05 compared with the NGF-treated group.

Activation of the FAK and c-Src pathways is an essential step in the progression of osteosarcoma 44. Stimulating osteosarcomas with NGF was shown to enhance FAK and c-Src phosphorylation in a time-dependent manner (Fig. 3A&B and 4A&B). Stimulating osteosarcoma cells with an FAK inhibitor or c-Src inhibitor (PP2) eliminated VCAM-1 synthesis, monocyte adhesion, and CD206 expression (Fig. 3C-E and 4C-E). Administration of the supernatant collected from these cells to THP-1 cells affected the expression of M2 macrophage marker CD206 mRNA expression (Fig. 3F and 4F). Transfecting osteosarcoma cells with FAK or c-Src siRNA was also shown to reduce VCAM-1 synthesis and monocyte adhesion (Fig. 3C-H and 4C-H). Stimulating osteosarcoma cells with an FAK inhibitor suppressed the NGF-facilitated phosphorylation of c-Src (Fig. 4I&J). These results suggest that FAK and c-Src pathway activation is involved in the NGF-mediated synthesis of VCAM-1 and monocyte adhesion.

**Figure 5:**
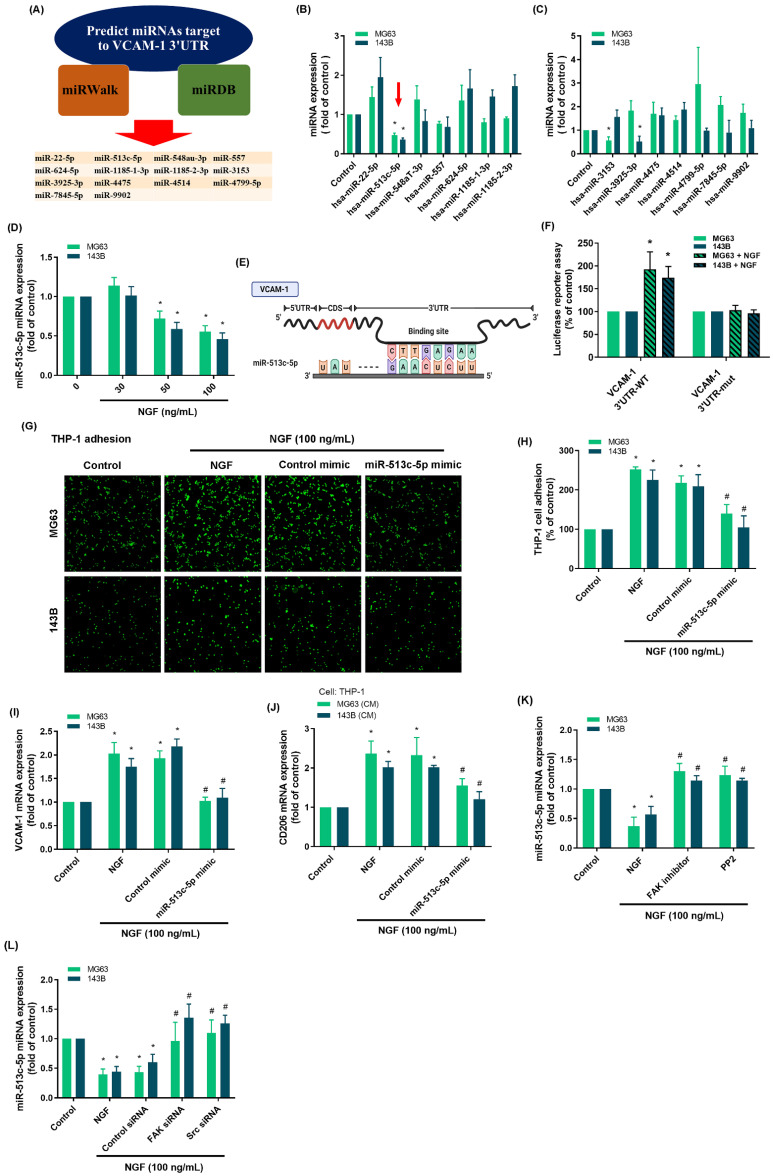
** NGF promotes VCAM-1 production and monocyte adhesion through the down-regulation of miR-513c-5p expression.** (A) miRNAs predicted to bind with VCAM-1 3'-UTR based on analysis of two miRNA prediction databases; (B-D) qPCR results showing miRNA expression after treating osteosarcoma cells with NGF; (E) miR-513c-5p binding site in VCAM-1 3'-UTR; (F) Osteosarcoma cells were transfected with the VCAM-1 3'-UTR wild-type or mutant plasmid for 24 h, then stimulated with NGF for 24 h, and relative luciferase activity was measured; (G-I) THP-1 adhesion and VCAM-1 expression in osteosarcomas transfected with miR-513c-5p mimic and then stimulated with NGF for 24 h; (J) The supernatant collected from osteosarcoma cells to THP-1 cells affected the expression of M2 macrophage marker CD206 mRNA expression; (K&L) miRNA expression in osteosarcoma cells incubated with FAK inhibitor and PP2 or transfected with FAK and c-Src siRNA and then stimulated with NGF for 24 h. All experiments were repeated at least three times. * *p* < 0.05 compared with the control group; # *p* < 0.05 compared with the NGF-treated group.

Researchers have compiled considerable evidence that miRNAs play crucial roles in the progression and apoptosis of cancer cells 45, 46. In the current study, we searched the miRWalk and miRDB online databases to identify miRNA targets within the 3'-UTR region of VCAM-1 mRNA (Fig. 5A). Among the 14 candidate miRNAs, miR-513c-5p presented the most pronounced down-regulation after NGF stimulation (Fig. 5B&C). Exposure to NGF markedly suppressed miR-513c-5p generation in two osteosarcoma cell lines in a concentration-dependent manner (Fig. 5D). The schematic diagram in Fig. 5E shows that the VCAM-1 3'-UTR contains a miR-513c-5p binding site. We investigated the impact of miR-513c-5p binding to the VCAM-1 3'-UTR, the results show that NGF was shown to enhance the luciferase activity of the wild-type VCAM-1 3'-UTR, whereas the mutant VCAM-1 3'-UTR generated no such response (Fig. 5F). Transfecting osteosarcoma cells with an miR-513c-5p mimic significantly reduced NGF-induced monocyte adhesion, VCAM-1, and CD206 expression (Fig. 5G-I). Administration of the supernatant collected from these cells to THP-1 cells affected the expression of M2 macrophage marker CD206 mRNA expression (Fig. 5J). Pre-treating osteosarcoma cells with FAK and c-Src inhibitors or their respective siRNAs blocked the effects of NGF in repressing miR-513c-5p expression (Fig. 5K&L). These results suggest that NGF augments VCAM-1-dependent monocyte adhesion by suppressing miR-513c-5p generation via the FAK and c-Src pathways.

### Larotrectinib blocked NGF-induced tumor growth of osteosarcoma cells

**Figure 6:**
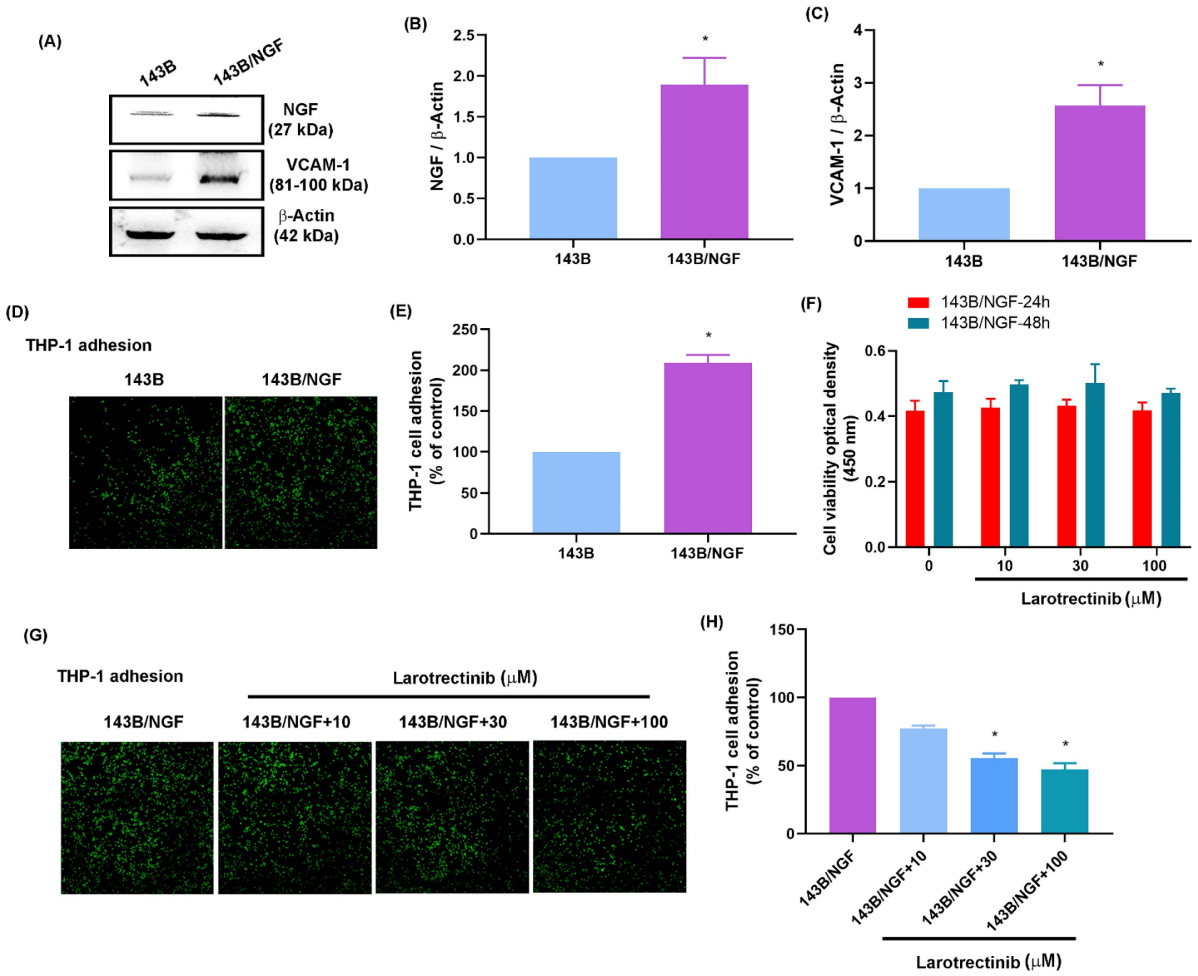
** Larotrectinib blocks NGF-induced monocyte adhesion to osteosarcomas.** (A-C) Western blot and qPCR results showing NGF and VCAM-1 expression in 143B and NGF-overexpressing (143B/NGF) cells; (D&E) Adhesion of THP-1 cells to 143B and 143B/NGF cells; (F) MTT assay results indicating viability of 143B/NGF cells incubated with larotrectinib for 24 or 48 h; (G&H) THP-1 adhesion in osteosarcoma cells incubated with larotrectinib and then stimulated with NGF for 24 h. All experiments were repeated at least three times. * *p* < 0.05 compared with the control group; # *p* < 0.05 compared with the NGF-treated group.

The effects of NGF were examined *in vivo* by generating an osteosarcoma cell line that overexpressed NGF (143B/NGF). It was found that 143B/NGF cells promoted NGF and VCAM-1 expression and monocyte adhesion (Fig. 6A-E). Larotrectinib is an orally administered ATP-competitive inhibitor of the TrK family, which blocks the expression of NGF 47. Cell viability assays demonstrated that larotrectinib (10 - 100 μM) did not have cytotoxic effects on 143B/NGF cells (Fig. 6F). Larotrectinib was also shown to suppress monocyte adhesion to 143B/NGF cells in a concentration-dependent manner (Fig. 6G&H).

**Figure 7:**
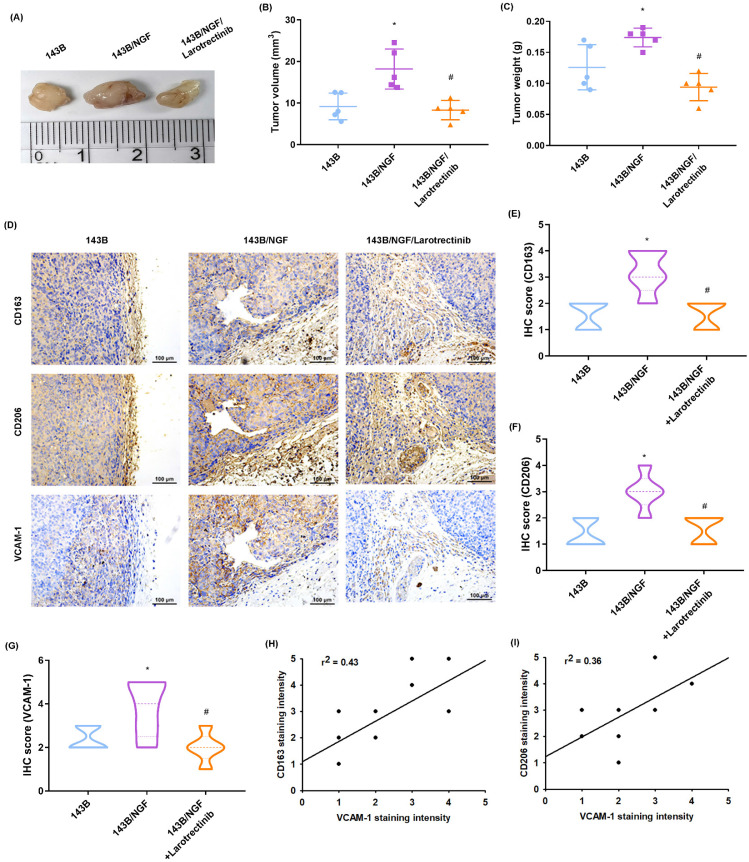
** Larotrectinib antagonizes NGF-induced osteosarcoma growth *in vivo*.** (A-C) Osteosarcoma cells were subcutaneously injected into the right flanks of mice. The 143B/NGF+Larotrectinib group was administered larotrectinib (50 mg/kg) orally three times a week. The tumor size and weight were measured after sacrificing the mice at four weeks; (D-G) IHC analysis indicating protein expression levels of CD163, CD206, and VCAM-1 in tumors; (H&I) Correlation between CD163 or CD206 versus VCAM-1 expression levels. All experiments were repeated at least three times. * *p* < 0.05 compared with the 143B group; # *p* < 0.05 compared with the 143B/NGF group.

In an *in vivo* xenograft model involving the subcutaneous injection of osteosarcoma cells into the right flank of mice, it was found that the resulting tumor volume and weight of the overexpressing NGF cell line (143B/NGF) were markedly greater than those of the 143B cell line (Fig. 7A-C). The oral administration of larotrectinib significantly inhibited NGF-mediated tumor growth (Fig. 7A-C). IHC staining revealed that NGF overexpression enhanced CD163, CD206, and VCAM-1 expression (Fig. 7D-G). We also observed that CD163 and CD206 were positively correlated with VCAM-1 expression levels (Fig. 7H&I). Moreover, larotrectinib inhibited the expression of CD163, CD206, and VCAM-1 (Fig. 7D-G). Taken together, these findings suggest that the NGF-promoted growth of osteosarcoma involves macrophage infiltration and M2 macrophages expression. Our findings also indicate that larotrectinib suppresses these effects.

## Discussion

Researchers are amassing considerable evidence of a correlation between cancer prognosis and tumor innervation, particularly in cancers with pronounced innervation, such as pancreatic, head and neck, prostate, and colorectal cancers 48. Analysis of patient tumor samples has revealed a positive correlation between tumor innervation density and the rates of metastasis, incidence, and mortality. Note that the extensive distribution of sensory nerves in bones often leads to CIBP in individuals with osteosarcoma 49. Researchers have also demonstrated that NGF secreted by tumors stimulates nerve regeneration and plays a key role in promoting the survival, motility, and invasion of tumor cells 50. Moreover, the knockout of Circ_0000006 potentiates the effects of doxorubicin on osteosarcoma-repressed cell proliferation, migration, and invasion via the miR-646/BDNF pathway 51. Exosomes have been implicated in the secretion of cellular hormones (including NGF) fostering an inflammatory and immunosuppressive microenvironment 48. Macrophages are highly adaptive cells capable of migration into and adaption to the cancer microenvironment. Several researchers have posited that tumors be viewed as complex microenvironments, rather than individual tumor cells 52. In the current study, we obtained *in vitro* and *in vivo* evidence of NGF facilitating VCAM-1-dependent monocyte adhesion and M2 macrophage polarization in the osteosarcoma microenvironment.

Receptor tyrosine kinases are important signaling molecules that regulate the proliferation, survival, differentiation, apoptosis, and migration of cells 53. The aberrant activation of tyrosine kinases has been linked to a variety of cancers. The activation of non-mutated kinases can lead to genomic alterations and contribute to the development and metastasis of various cancers, particularly those with a low mutation rate (e.g., prostate cancer).

Researchers have identified several tyrosine kinase inhibitors (TKIs) with varying degrees of activity against TRKA, TRKB, and/or TRKC. These TKIs can be broadly classified as multi-kinase inhibitors with activity against a range of targets, including TRK or more selective TRK inhibitors 53. Multi-kinase inhibitors include entrectinib, crizotinib, cabozantinib, lestaurtinib, altiratinib, foretinib, ponatinib, nintedanib, merestinib, MGCD516, PLX7486, DS-6051b, and TSR-011 54. First-generation tropomyosin receptor kinase (TrK) inhibitors (larotrectinib and entrectinib) act as ATP-competitive inhibitors specifically binding to the ATP binding site in the active conformation of their target enzyme (DFG-in) 53. Currently, larotrectinib is the most specific TrK family inhibitor undergoing assessment for the treatment of cancer. It is also a novel candidate for targeting the NGF-mediated progression of osteosarcoma.

The tumor microenvironment plays an important role in the development and spread of tumors. Recent research has revealed that the infiltration of inflammatory cells into the stroma surrounding the tumor is crucial to its growth 55, 56. Macrophages are the most prevalent inflammatory cells in the tumor environment. Previous research has demonstrated that macrophages can easily penetrate the borders of tumors 57, 58. In the current study, our aim was to elucidate the means by which NGF enhances monocyte adhesion in the osteosarcoma microenvironment. The GDC data portal revealed elevated levels of M1 macrophage markers (CD163 and CD206) without any M2 macrophage markers in osteosarcoma samples. The overexpression of NGF has been shown to promote tumor growth and the expression of M2 macrophage markers *in vivo*. Our data suggest that the effects of NGF in facilitating the growth of osteosarcoma involve the expression of M2 macrophage markers.

FAK-induced c-Src pathway activation has been shown to play a crucial role in mediating various cellular activities 59, 60. In the current study, we discovered that FAK and c-Src inhibitors moderated the effects of NGF in promoting VCAM-1 production as well as monocyte adhesion. Genetic inhibition induced by FAK and c-Src siRNAs yielded similar effects. Incubating osteosarcoma cells with NGF was shown to augment FAK and c-Src phosphorylation. The fact that FAK inhibitors reversed NGF-enhanced c-Src phosphorylation suggests that in human osteosarcoma, NGF activates the FAK and c-Src signaling pathways and in so doing promotes VCAM-1-regulated monocyte adhesion. Multiple lines of evidence highlight the significance of the FAK and c-Src pathways in tumor functions. For instance, amphiregulin induces VEGF-A synthesis and angiogenesis in human chondrosarcoma via the FAK/c-Src pathway 61. Moreover, D-pinitol interferes with the motility of prostate cancer cells by suppressing FAK and c-Src cascades 62. Finally, NGF has been shown to facilitate chondrosarcoma metastasis through the activation of FAK/c-Src signaling 29.

miRNAs have been implicated in a number of diseases, such as cancer, obesity, cardiovascular diseases, and diabetes 63, 64, and recent research has demonstrated that miRNAs play key roles in the cancer microenvironment. For example, miR-30a antagonists inhibit LOX expression and affect thyroid cancer differentiation 65. Moreover, the expression of miR-141-3p and miR-145-5p has been shown to suppress the migration and invasion of renal cell carcinoma 66. In chondrosarcoma, miR-27b and miR-519d are regulated by adipocytokines 15, 16, 51. Bioinformatics analysis in this study identified VCAM-1 as a direct target of miR-513c-5p. This was the first study to demonstrate a correlation between the miR-513c-5p/VCAM-1 axis and macrophages in osteosarcoma. We demonstrated that miR-513c-5p expression was profoundly down-regulated after NGF treatment and that an miR-513c-5p mimic eliminated the NGF-mediated synthesis of VCAM-1 and cell monocyte adhesion. Moreover, the inhibition of FAK and c-Src countered the decrease in miR-513c-5p synthesis induced by NGF, which suggests that NGF modulates VCAM-1-dependent monocyte adhesion to osteosarcoma cells by inhibiting miR-513c-5p levels via the FAK and c-Src pathways.

Experiments in the current study confirmed that the oral administration of the TrK inhibitor larotrectinib significantly inhibited the effects of NGF on M2 macrophage levels and osteosarcoma progression; however, it is important to consider the complexity of the *in vivo* tumor microenvironment. Further research will be required to elucidate the effects of larotrectinib on other cells associated with osteosarcoma progression. Moreover, researchers will have to overcome various challenges in the use of molecular therapy to target TrK inhibitors. It will be necessary to identify selective inhibitors and assess their efficacy in preclinical and clinical studies. It will also be necessary to develop effective delivery methods for the transport of these compounds to target cells. Nonetheless, the targeting of TrK inhibitors remains a promising therapeutic strategy for various diseases, including osteosarcoma.

**Figure 8:**
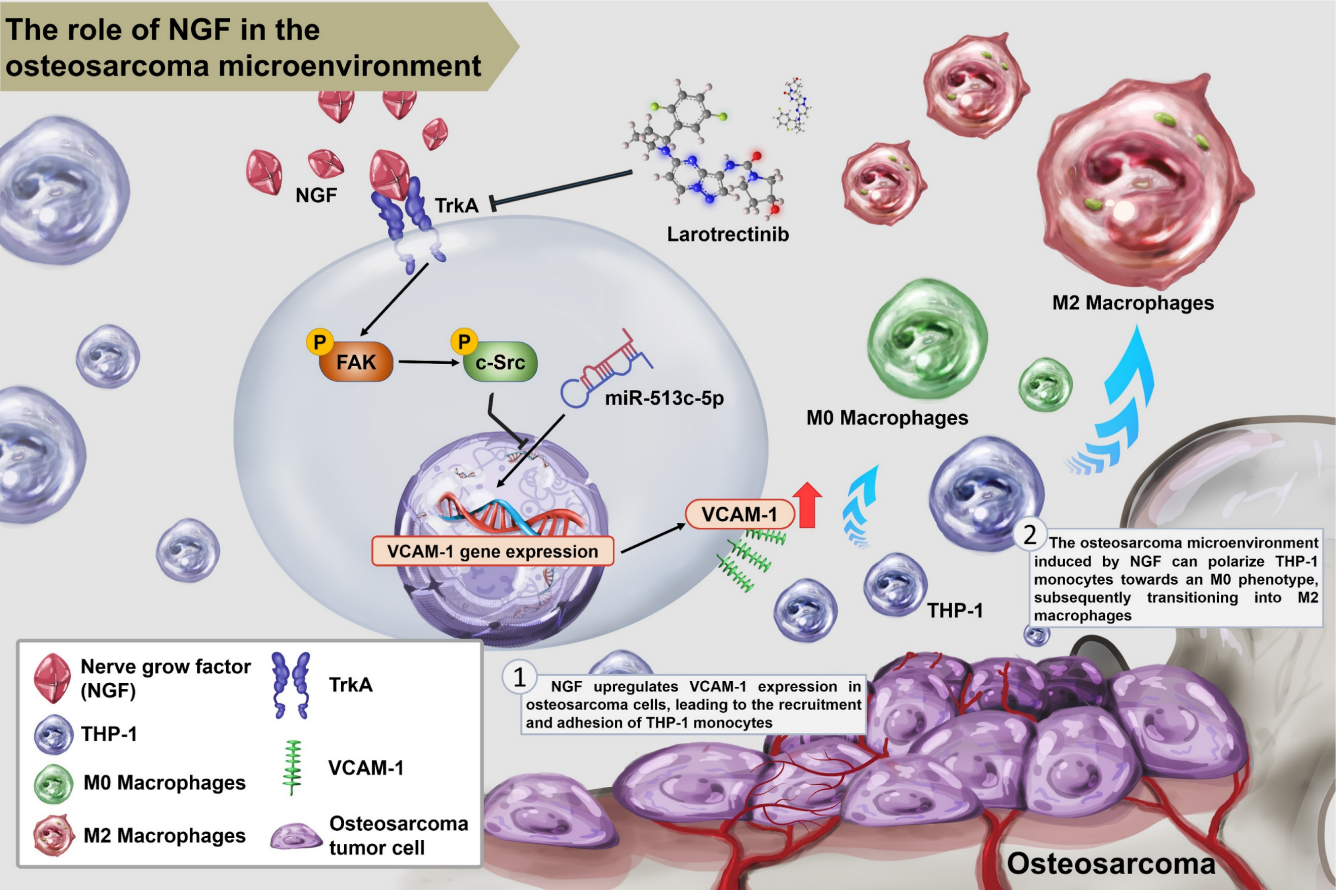
** Schematic diagram illustrating the mechanism underlying the effects of NGF in an osteosarcoma microenvironment.** NGF facilitates VCAM-1-dependent monocyte invasion into the osteosarcoma microenvironment and then enhances M2 macrophage polarization by inhibiting miR-513c-5p levels through the FAK and c-Src signaling cascades. Larotrectinib effectively suppresses NGF-induced cell growth.

In summary, this study demonstrated that NGF augments VCAM-1-dependent monocyte adhesion within the osteosarcoma microenvironment and facilitates M2 macrophage polarization by inhibiting miR-513c-5p expression via the FAK and c-Src signaling cascades. Larotrectinib presented anti-tumor effects through the inhibition of NGF-promoted tumor growth (Fig. 8).

## Figures and Tables

**Table 1:** List of PCR and primer used for the experiments

Target mRNA	Forward primer (5'→3')	Reverse primer (5'→3')
**CD14**	GACCTAAAGATAACCGGCACC	GCAATGCTCAGTACCTTGAGG
**CD68**	GGAAATGCCACGGTTCATCCA	TGGGGTTCAGTACAGAGATGC
**CD80**	AAACTCGCATCTACTGGCAAA	GGTTCTTGTACTCGGGCCATA
**IL-1b**	TTCGACACATGGGATAACGAGG	TTTTTGCTGTGAGTCCCGGAG
**CD163**	GCGGGAGAGTGGAAGTGAAAG	GTTACAAATCACAGAGACCGCT
**CD206**	GGGTTGCTATCACTCTCTAGC	TTTCTTGTCTGTTGCCGTAGTT
**VCAM-1**	TTTGACAGGCTGGAGATAGACT	TCAATGTGTAATTTAGCTCGGCA
**GAPDH**	ACCACAGTCCATGCCATCAC	TCCACCACCCTGTTGCTGTA
